# Endemicity, Clinical Features, Risk Factors, and the Potential for Severe Infection in *Leptospira wolffii*-Associated Leptospirosis in North-Central Bangladesh

**DOI:** 10.3390/tropicalmed10100290

**Published:** 2025-10-13

**Authors:** Sheikh Anika Tasnim, Nazia Haque, Shyamal Kumar Paul, Meiji Soe Aung, Md. Rafiul Hasan, Sheikh Nayeem Niaz, Arup Islam, Syeda Anjuman Nasreen, Mosammat Rezaun Nahar, Sultana Jahan Tuly, Parsa Irin Disha, Abdullah Al Mamun, Md. Shafiqul Islam, Santana Rani Sarkar, Nobumichi Kobayashi

**Affiliations:** 1 Department of Microbiology, Mymensingh Medical College, Mymensingh 2200, Bangladesh; sheikhanikatasnim59@gmail.com (S.A.T.); drnaziahaque@gmail.com (N.H.); drarup@gmail.com (A.I.); nasreenm19@gmail.com (S.A.N.); urmi4rakib@gmail.com (M.R.N.); sultanatuly15@gmail.com (S.J.T.); dishaparsairin@gmail.com (P.I.D.); aamamun6985@gmail.com (A.A.M.); 2 Department of Microbiology, International Medical College, Gazipur 2000, Bangladesh; drshyamal10@yahoo.com; 3 Department of Social Medicine, Division of Hygiene, Sapporo Medical University, Sapporo 060-8556, Japan; meijisoeaung@sapmed.ac.jp; 4 Directorate General of Health Services, Dhaka 1212, Bangladesh; dr35rafi@gmail.com; 5 Shaheed Suhrawardy Medical College, Dhaka 1207, Bangladesh; sknayeemniaz10@gmail.com; 6 Department of Microbiology and Hygiene, Faculty of Veterinary Science, Bangladesh Agricultural University, Mymensingh 2202, Bangladesh; shafiq_micro@bau.edu.bd; 7 Department of Microbiology, Netrokona Medical College, Netrokona 2400, Bangladesh; dr.santanasarkar@gmail.com

**Keywords:** *Leptospira wolffii*, 16S rRNA, P2 subclade, IgM ELISA, nested PCR, Bangladesh

## Abstract

Leptospirosis is a zoonotic disease caused by pathogenic *Leptospira*, prevalent in tropical/sub-tropical regions. This study aimed to clarify the prevailing leptospiral species, clinical features, and risk factors of leptospirosis in north-central Bangladesh in 2024. Venous blood and urine samples were collected from 117 patients with clinically suspected leptospirosis. Among these cases, 75 (64%) tested positive for *Leptospira* infection by IgM ELISA test and/or PCR. By phylogenetic analysis of the 16S rRNA gene, all the samples tested were classified into *L. wolffii* (pathogenic group P2), showing high sequence identity to those of the type strain Khorat-H2 (97–99%) and *L. wolffii* reported in Bangladesh previously. Confirmed leptospirosis patients were mostly male (93%), aged 15–60 years (93%), living in rural areas in low socioeconomic conditions. Variable symptoms were presented by patients, with jaundice (84%), nausea/vomiting (84%), and myalgia (67%) being common. Some patients showed severe symptoms involving the nervous system (disorientation and neck stiffness) and the respiratory tract (cough, shortness of breath, and hemoptysis). Major risk factors for leptospirosis were exposures to mud/wet soil, sanding water, heavy rain, working in a paddy field, and cattle. In conclusion, *L. wolffii* was revealed to be circulating endemically in north-central Bangladesh, since its first detection in 2018, associated with variable and severe clinical symptoms in humans.

## 1. Introduction

Leptospirosis is a reemerging, neglected zoonotic infectious disease caused by spirochetes of the genus *Leptospira*. This disease is widespread globally, except in Antarctica, with frequent outbreaks in regions such as the Indian subcontinent, Oceania, and Latin America [1], and is of particular concern in tropical and subtropical regions, including Bangladesh, posing a significant public health threat [2]. It was estimated that there are approximately 1.03 million cases of leptospirosis globally every year, resulting in 58,900 deaths [3]. Median series mortality is shown as 2.2% (0–40%) among untreated cases of leptospirosis, while higher mortality is noted for patients with jaundice, renal failure, and those aged over 60 years [4]. Environmental factors, urbanization, inadequate sanitation, and climate change-induced flooding are expected to exacerbate the disease burden [5]. Despite its high prevalence, leptospirosis remains underdiagnosed and underreported due to its protean clinical manifestations, lack of awareness among healthcare providers, limited diagnostic facilities in poor-resource settings, and the absence of sensitive tests for early-stage detection [5,6].

The transmission cycle of *Leptospira* involves reservoir hosts (livestock, pets, rodents, and wildlife), environmental factors, and humans. *Leptospira* is widely distributed to numerous mammalian species including cattle, horse, pig, dog, camels, and rodents [7,8], as well as amphibia and reptilia [9], indicating a global risk of leptospirosis in humans. In these animals, leptospires circulate in the bloodstream and colonize the brush border of the proximal renal tubular epithelium via glomerular or peritubular capillaries. Pathogenic *Leptospira* are excreted into the environment via urine from reservoir hosts which are asymptomatic carriers. Humans are infected with leptospires through contact with the contaminated environment [5].

The genus *Leptospira* comprises 68 species, classified into two primary clades: S (saprophytes) and P (pathogens). These clades are further subdivided into four subclades: S1 and S2 (saprophyte subclades), P1 (formerly categorized as the pathogen group), and P2 (previously referred to as the “intermediate” group) [10]. Saprophytic *Leptospira* species, belonging to the S clade, are free-living spirochetes commonly found in soil and surface water, and they typically do not cause disease in mammals. In contrast, the P1 subclade includes species with high pathogenicity, which are capable of infecting a wide range of animal species and are transmissible to humans. The P2 subclade contains species exhibiting moderate pathogenicity, affecting both humans and animals [11]. These species are further designated into hundreds of serovars, which are grouped into serogroups based on antigenic relatedness [12].

In Bangladesh, leptospirosis was shown to be prevalent, especially in flood-prone rural areas, due to risk factors such as a tropical climate, inadequate sanitation, frequent flooding, many animal reservoirs, and an agriculture-based economy which significantly increases the risk of leptospirosis [2,13]. Serological testing indicated that leptospirosis accounts for 8.6% of outpatient fever cases [13], 10.6% of healthy individuals in rural areas, and 12.0% in the urban febrile population [14]. Since 2019, the Institute of Epidemiology, Disease Control and Research (IEDCR), Bangladesh, has been conducting a nationwide surveillance of leptospirosis across eight sentinel sites by detection of IgM antibodies and confirmatory PCR testing (https://iedcr.portal.gov.bd/site/page/4dd56857-84df-4269-b341-04e9e8a55725, accessed on 14 March 2025). Between 2019 and 2023, 4.4% to 8.3% of clinically suspected cases (a total of 5325 samples) were reported to be positive for *Leptospira* (IEDCR, 2024). Compared with these findings, relatively higher detection rates of *Leptospira* by the genetic method were revealed in Mymensingh, the north-central region of Bangladesh, in 2018 (17.6%) and 2019 (35.7%), showing the dominant species as *L. interrogans* and *L. wolffii*, respectively [15,16]. Thereafter, between 2021 and 2022, leptospirosis accounted for 47% among suspected cases (n = 186), with the detection of only *L. wolffii* [17]. The predominance of *L. wolffii* was notable because it belongs to the P2 subclade and its pathogenicity and epidemiological features have been rarely described in this region.

The present study aimed to clarify the prevalence of leptospirosis and prevailing leptospiral species in north-central Bangladesh, to monitor the persistence or change of dominant species since the initial study in 2018. In addition, clinical features and risk factors of leptospirosis that might have been associated with the dominant species were investigated to explore further preventive measures.

## 2. Materials and Methods

### 2.1. Collection of Specimens

**Figure 1:**
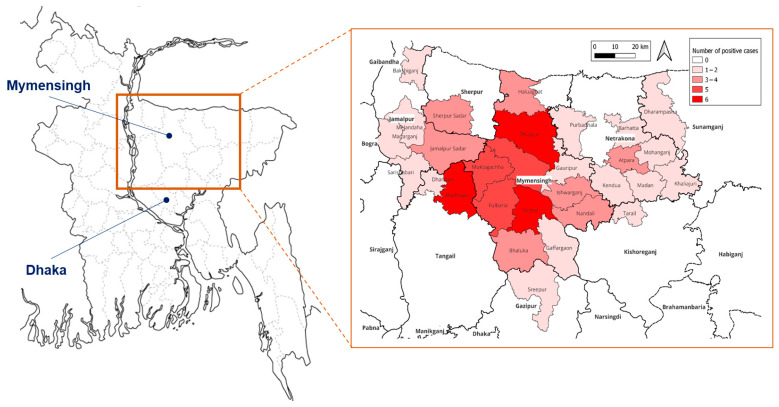
Location of Mymensingh in Bangladesh (left) and incidence of leptospirosis patients in each place of residence (extracted map in a square).

This study was conducted as a cross-sectional, observational study. Venous blood and urine samples were collected from patients with clinically suspected leptospirosis who visited Mymensingh Medical College Hospital in Mymensingh city and agreed to participate in this study, for a period from March 2024 to February 2025. Mymensingh is a central city in Mymensingh Division, located about 100 km north of the capital city Dhaka (Figure 1). Inclusion criteria for cases were as follows: (1) fever for more than five days with or without malaise, headache, myalgia, anorexia, abdominal pain, or conjunctival suffusion, (2) febrile patients with any of the symptoms of hepatic involvement such as jaundice, renal involvement such as oliguria and hematuria, and pulmonary symptoms such as cough and hemoptysis. Exclusion criteria were (1) patients suffering from a febrile illness where any underlying etiology other than leptospiral illness had already been established, and (2) patients who did not give written informed consent. The blood sample (5 mL) was collected aseptically, placed in a sterile test tube, and allowed to stand for 30 min to facilitate clot formation, followed by centrifuging at 3000 rpm for 5 min. The serum was separated and transferred into a sterile tube for ELISA and polymerase chain reaction (PCR) analysis.

### 2.2. Detection of Leptospira-Specific IgM by ELISA

*Leptospira*-specific IgM in serum is detectable from day 5 onwards after symptom onset and usually persists for several months. Detection of the IgM antibodies was performed using the Panbio™ Leptospira IgM ELISA kit (Abbott, Chicago, IL, USA) [18] following the manufacturer’s protocol.

### 2.3. Urine Culture for Leptospira

In total, 500 µL of the urine sample was inoculated into semi-solid Ellinghausen and McCullough modified by Johnson and Harris (EMJH) media supplemented with 2% bovine serum albumin and incubated at 30 °C [19]. The cultures were maintained for up to 2 months with regular examinations for growth. For any presumptive positive culture, two serial subcultures were performed every 7–14 days to ensure bacterial growth. The growth of *Leptospira* in the cultures was examined weekly using dark-field microscopy.

### 2.4. Detection of Leptospira by PCR

From the serum sample, genomic DNA was extracted according to a method described previously [20]. Genomic DNA in the serum sample was extracted using the phenol–chloroform method. From urine sample, the direct lysis method described previously [21] was employed for DNA extraction. For the culture sample, the pellet of culture fluid after centrifugation was suspended in 20 µL of 10 mM Tris-1 mM EDTA (TE; pH 8.0). After boiling, supernatant followed by centrifugation was used as the DNA sample. Nested PCR, targeting 16S rRNA of *Leptospira* including the pathogenic and saprophytic clades, was performed by using the protocol and primers described previously [20] for serum and urine samples, and cultured samples. As a thermostable DNA polymerase for PCR, TaKaRa Ex Taq^®^ (Takara, BIO INC, Kusatsu, Japan) was used. The second-round PCR product was electrophoresed on agarose gel and visualized as a band using a gel documentation system. In addition, conventional PCR to detect the pathogenic species of *Leptospira* targeting *lig* gene was performed as described previously [22] for genus-specific PCR-positive samples. Double-distilled water was used as a negative control. Though positive control was not available, the PCR products in this study were sequenced to confirm its derivation from the target gene, as shown below.

### 2.5. Sequencing and Phylogenetic Analysis

Species of *Leptospira* was identified by determination of the nucleotide sequence of the partial 16S rRNA by Sanger sequencing with the PCR product (289 bp) using a BigDye Terminator v3.1 Cycle sequencing kit (Applied Biosystems, Foster City, CA, USA) on an automated sequencer (Applied Biosystems^®^ PRISM3130 Genetic Analyzer, Thermo Fisher Scientific, Foster City, CA, USA). The sequence data obtained were analyzed using BLAST on the NCBI website (https://blast.ncbi.nlm.nih.gov/Blast.cgi, accessed on 15 March 2025), to search for the most similar sequence in the GenBank database. The Clustal Omega program (https://www.ebi.ac.uk/jdispatcher/msa/clustalo, accessed on 15 March 2025) was used to perform the multiple nucleotide alignment and obtain sequence identity. A phylogenetic dendrogram of the 16S rRNA was constructed with the maximum likelihood method using the MEGA ver.6 software package, together with sequences of representative *Leptospira* strains in GenBank database. The representative sequence data of *Leptospira* samples in this study were deposited in the GenBank under the accession numbers PV367219-PV367223, PV367229, and PV367230.

### 2.6. Patients’ Information, Risk Factors, and Statistical Analysis

Clinical symptoms of the individual patients, their sociodemographic factors, and risk factors were recorded on data sheets, together with the laboratory test results. The difference in the variables between different patient groups was statistically analyzed with Fisher’s exact test using the js-STAR XR ver.1.1.9 software (https://www.kisnet.or.jp/nappa/software/star/index.htm, accessed on 10 April 2025). The difference in the *Leptospira*-positive rates by the different methods was analyzed with McNemar’s test. A *p*-value < 0.05 was considered to be statistically significant.

## 3. Results

### 3.1. Detection of Leptospira by ELISA, PCR, and Culture

**Figure 2:**
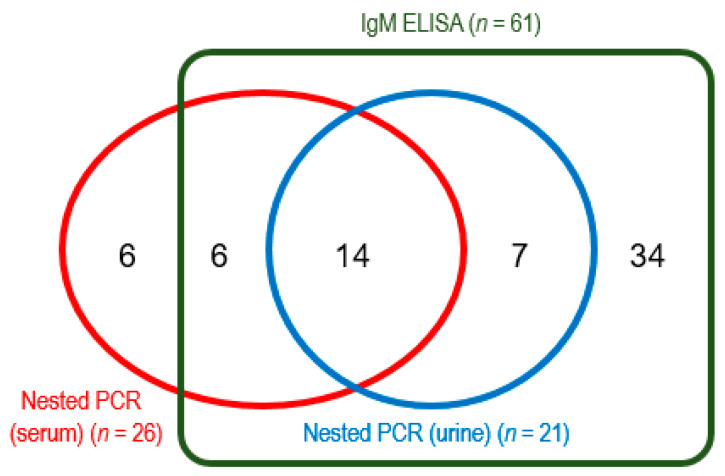
Number of positive samples by IgM ELISA and nested PCR for urine and serum, among 88 samples. The remaining 29 samples were tested only by PCR (8 samples were positive).

During the study period, 117 clinically suspected cases of leptospirosis were selected by the criteria. Of these patients, 88 were tested by both IgM ELISA and nested PCR (urine and serum), and the remaining 29 were examined only by nested PCR for serum samples. Positive rate of *Leptospira*-specific IgM by ELISA was 69.3% (61/88), which was higher than those by nested PCR for serum samples (29.1%, 34/117) and urine samples (23.9%, 21/88). Among a total of 117 cases, 75 (64%) were tested positive for *Leptospira* infection by any of the diagnostic methods. All the PCR-positive urine samples and most of the serum samples (20 among 26) were IgM-positive (Figure 2). However, 39% (34/88) and 7% (6/88) of samples were positive in only ELISA and PCR for serum, respectively.

The nested PCR for serum and urine showed a higher detection rate among cases with a shorter period of fever (5–7 days) than those with a longer period (8 or more days) (Table 1). In contrast, in cases with the longer fever periods, IgM-ELISA-positive rates were significantly higher than those by nested PCR for serum. Culture of *Leptospira* was attempted for 20 PCR-positive urine samples. The growth of *Leptospira* was confirmed in four samples, among which three samples were derived from cases with 8–14-day duration of fever (Table 1).

### 3.2. Genetic Analysis of 16S rRNA and lig Gene

**Figure 3:**
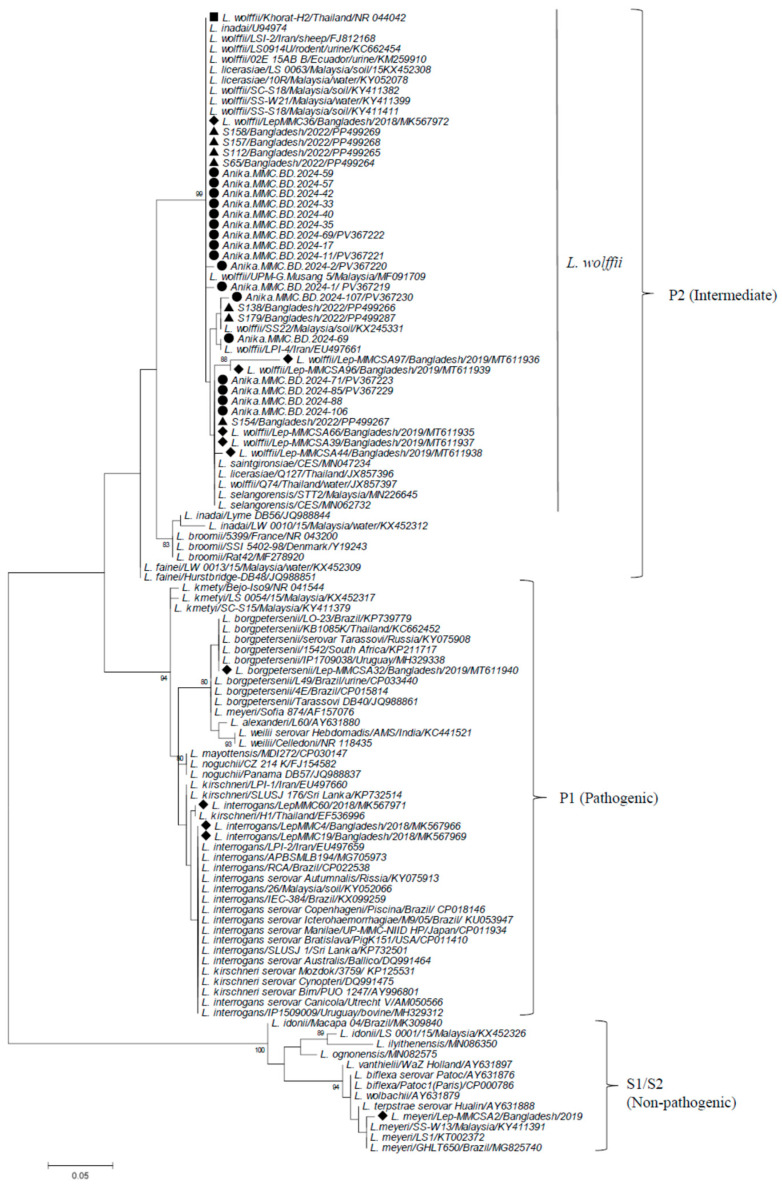
Phylogenetic dendrogram based on partial 16S rRNA gene sequences of *Leptospira* constructed by the maximum likelihood method using the MEGA6 program, following alignment with the ClustalW algorithm. The trees were statistically supported by bootstrapping with 1000 replicates, and phylogenetic distances were measured by the Kimura 2-parameter model with uniform rates among sites. Samples analyzed in the present study are marked with closed circles, while those in the previous studies in Mymensingh, Bangladesh, are shown with triangles (2022) and diamonds (2018–2016), respectively. *L. wolffii* prototype strain Khorat-H2 is indicated by a closed square. Bootstrap values more than 80% are shown. The scale bar represents the genetic distance. Subclusters are shown on the right using the designations by Guglielmini et al. (P1, P2, and S1/S2) [24] with former designations (pathogenic, intermediate, and non-pathogenic). A cluster of *L. wolffii* and closely related species to *L. wolffii* in the P2 subclade are shown by a vertical line.

Partial 16S rRNA sequences were determined for the selected 17 samples (13 serum and 4 urine culture samples) obtained from July to October 2024 (Table S1). Among these samples, sequence identity was 98.6–100% (Table S2). Phylogenetic analysis revealed that all the samples belonged to *L. wolffii* in the P2 subclade, clustering with *L. wolffii* type strain Khorat-H2 [23] and those reported in Bangladesh previously [15,16,17], showing sequence identity of 95–100% (97–99% with Khorat-H2 strain) (Figure 3 and Table S3). A specific amplicon was not obtained from any 16S rRNA-positive samples in the PCR targeting *lig* gene.

### 3.3. Sociodemographics, Clinical Findings, and Risk Factors of Leptospirosis

Confirmed leptospirosis patients were mostly male (93%), aged 15–60 years (93%), and lived in rural area (92%), in low socioeconomic conditions (poor) (91%), with half of the patients being farmers (Table 2). The peak of leptospirosis cases was found in September, the end of the rainy (monsoon) season, though cases occurred throughout the year. Geographically, patients’ residences were distributed across Mymensingh district and neighboring regions, with higher case concentrations (5–6 patients) observed particularly in Trishal and Fulbaria upazilas of Mymensingh district, and Madhupur upazila of the adjoining Tangail district (Figure 1).

Clinical symptoms of leptospirosis patients were variable, as shown in Table 3, though their incidence rates were not statistically compared with non-leptospirosis cases. Malaise (96%) and jaundice (84%) were most prominent, with also frequent incidence of myalgia (66.7%), and manifestations of gastrointestinal tract (e.g., nausea/vomiting) and musculoskeletal system (e.g., calf muscle pain). Some patients presented severe symptoms involving nervous systems (e.g., disorientation and neck stiffness) and respiratory tract (e.g., shortness of breath and hemoptysis). Leukocytosis associated with thrombocytopenia, low hemoglobin, and raised bilirubin/creatinine/ALT were commonly seen in patients (>60%), with high incidence of pus cells in urine (82.9%) (Table 3). In agreement with the laboratory findings, nephritis and hepatitis were indicated by ultrasonography for suspected cases (63% and 29%, respectively). Additionally, evidence of plural effusion was found in the four leptospirosis patients by chest X-ray and High-Resolution Computed Tomography (HRCT), indicating alveolar hemorrhage in one case.

Among the leptospirosis cases, rural patients had high frequencies of exposure to mud or wet soil (78.3%) and standing water (75.4%), often due to activities like working in paddy fields (56.5%), fishing (39.1%), and swimming (33.3%). They also had significant animal exposure, particularly to cattle (75.4%), dogs (73.9%), and rodents (65.2%). Both rural and urban patients were mostly affected by heavy rain, while involvement of wound and travel history was less prominent (Table 4).

## 4. Discussion

Leptospirosis is one of the major globally widespread zoonotic diseases, causing a presumptive high disease burden in developing countries. In Bangladesh, a high seroprevalence of *Leptospira* (38%) evaluated by the microscopic agglutination test (MAT) was reported in a rural flood-prone area in the early 1990s [2]. Thereafter, in Dhaka (2001), the prevalence of leptospirosis diagnosed among febrile patients was reported as 18% during a dengue outbreak [25], and 8.4% among febrile patients in a low-income area [13]. In Mymensingh, a city of north-central Bangladesh, leptospirosis was diagnosed by serological tests and PCR in 17.6–47% of suspected febrile patients from 2018 to 2022 [15,16,17]. In the present study conducted in the same study site/hospital (March 2024–February 2025), 64% of suspected cases examined were diagnosed as leptospirosis by PCR or IgM ELISA (PCR-positive rate, 29.1%). Though detection rates might be affected by the number of cases, differences in detection methods, and selection of cases via differential diagnosis, a series of studies in Mymensingh indicated that leptospirosis has been endemically persisting at high prevalence as a febrile disease in this region.

In South Asia and Southeast Asia, *L. interrogans* has been described as the most common species, as summarized in Table 5. Other species often identified are *L. borgpetersenii*, *L. kirschneri*, and *L. weilii*, though *L. wolffii* has been rarely reported. However, in our present study, only *L. wolffii* was identified from samples selected throughout the study period. In Mymensingh, this species was first reported in only one sample with other common species (*L. interrogans*) in 2018 [15]. Thereafter, *L wolffii* was predominantly detected in 2019 [16], with the sole detection of *L. wolffii* from 2021 until 2024 [17], including our present study. This suggests that *L. wolffii* has been endemic to north-central Bangladesh, at least since 2019. *L. wolffii* was assigned as a new species for the type strain Khorat-H2 from a patient in Thailand [23]. *L. wolffii* was originally classified into a group with intermediate pathogenicity [26] and genetically assigned to pathogenic subclade P2 [10], which was further grouped into P2-2 subgroup as a sole member, distinct from the other two subgroups P2-1 (e.g., *L. fainei*) and P2-3 (e.g., *L. licarasiae*) [24]. Detection of *L. wolffii* from humans was reported in Iran, including a study with its dominance [27,28], India [29], Malaysia [30], Ecuador [31], and Argentina [32]. Recently, in northeastern India (Sikkim and Assam) located near Bangladesh, a fatal case of *L. wolffii* infection [33] and an outbreak of leptospirosis due to *L. wolffii* were reported [34]. This species has also been detected in animals (cow, goat, buffalo, dog, raccoon dog, and Eurasian otter) in Iran [28], India [29], and South Korea [35]. Furthermore, *L. wolffii* DNA was detected in environmental water and soil in Malaysia [36,37,38], Thailand [39,40], and a southern island in Japan [41]. These findings suggest that *L. wolffii* may be distributed to humans and animals primarily in a wide region from eastern to western Asia. Although its incidence in humans with leptospirosis appears to be low, except in Bangladesh (Mymensingh) and Iran, monitoring of *L. wolffii* may be necessary in Asia due to its potential for widespread distribution in the environment.

Little is known about the clinical features of leptospirosis due to *L. wolffii*, as well as other *Leptospira* previously referred to as “intermediate pathogenic group”, as it has been just described as being associated with mild disease [52] or minimal/no disease [26]. In the present study in 2024 and the previous study in 2021–2022 [17] in the same study site in Bangladesh, only *L wolffii* was identified, though not all the samples could be analyzed. Accordingly, clinical and demographic findings on cases obtained from these studies are deemed to generally represent those due to *L. wolffii* infection. In these studies, jaundice, nausea/vomiting, and myalgia were found in 66–84% of cases, with 15% having conjunctival suffusion. In contrast, among leptospirosis outbreaks worldwide, jaundice and myalgia were found in about 50% and 4%, respectively [53]. In the early studies in Bangladesh (2001), jaundice and conjunctival suffusion were rare (5% and 2%, respectively), while myalgia was common (67–85%) [13,25]. In a study in Malaysia where *L. interrogans* and *L kirschneri* (P1) were dominant, gastrointestinal signs (59%) and respiratory signs (46%) were common, with myalgia being less frequent (30%) [30]. In our present study, gastrointestinal symptoms (nausea/vomiting, diarrhea, and abdominal pain) were also frequently reported, along with respiratory symptoms such as cough and shortness of breath. Relevant laboratory tests revealed the high incidence of elevated serum bilirubin, creatinine, thrombocytopenia, and leukocytosis, with the ultrasonographic findings of nephritis, hepatitis, and ascites indicating a severe systemic infection with multi-organ involvement. Hemoptysis was present in three patients; among them, one patient showed evidence of pulmonary hemorrhage in HRCT, suggesting severe pulmonary hemorrhagic syndrome (SPHS), a serious complication of leptospirosis. These findings suggest that *L. wolffii* may be responsible for severe diseases comparable to leptospirosis caused by pathogenic *Leptospira*. For other intermediate pathogenic species, available information is limited, though mild to severe cases were reported for *L. fainei*, *L. liceraciae*, and *L. venezuelensis* [54,55,56]. Phylogenetically, *L. wolffii* is somewhat distinct from other species among the P2 subclade [24], with an almost similar profile of virulence factors [57]. Therefore, among the P2 subclade, *L. wolffii* is suggested to have high pathogenicity to humans, similar to the leptospiral species of the P1 subclade. We observed higher rates of genetic and serological detection of *Leptospira* in the early and late stages of disease, respectively. This was also described previously [17,58], being in line with the kinetics of leptospiral cells and antibody in the blood of patients [59], suggesting that the immune response to *Leptospira* is common irrespective of its species.

In the present study, the peak incidence of leptospirosis was observed in the end of rainy season (September), which had been similarly reported in Thailand [60] and India [61]. Such seasonality of leptospirosis may be related to the risk factors of the disease found in the present study, exposure to standing water, mud/wet soil, and contact with rodents and cattle, which have also been described as environmental factors of this disease [62]. *L. wolffii*, which was dominant in human cases in this study, has been commonly detected in various animal species, environmental water, and soil, as described above. Therefore, for prevention of leptospirosis in north-central Bangladesh, reducing the opportunity to come into contact with the risk factors may be primarily important, particularly in rural areas during the rainy season.

The present study has some limitations. Due to a limited study period, the number of suspected cases was relatively smaller for more accurate evaluation of the prevalence, clinical features, and risk factors of leptospirosis in the study site. Because sufficient amount of blood samples was not obtained from some patients, serum was used for nested PCR as well as IgM ELISA for all the subjects, and positive controls of target genes were not available, which might lead to a potential underestimation of positive cases. However, our present and previous studies [16,17] strongly suggested the predominance of *L. wolffii* belonging to the P2 subclade and its involvement in severe symptoms in humans in north-central Bangladesh. Accordingly, further studies may be required to determine the prevalence of *L. wolffii* in other divisions/regions in Bangladesh as well as South Asian countries, and its relevance to clinical symptoms in human cases. In addition, studying the possible distribution of *L. wolffii* in animal species and the environment could be useful to devise preventive measures for leptospirosis in the endemic areas.

## 5. Conclusions

In conclusion, the present study revealed the high prevalence of leptospirosis in north-central Bangladesh due to *L. wolffii* (P2 subclade), which may be persisting endemically as a dominant species. Furthermore, the probable association of *L. wolffii* with severe symptoms was suggested. These observations underscore the importance of P2 subclade *Leptospira* as a significant and probably emerging cause of human leptospirosis. The risk factors of *L. wolffii*-associated leptospirosis were generally related to a contaminated environment, as observed for other virulent *Leptospira*. Therefore, for the control of leptospirosis in Bangladesh, further surveillance and clinical studies are necessary, as well as improvement of the hygienic conditions of the population.

## Figures and Tables

**Table 1:** Diagnosis of leptospirosis by different detection methods and duration of fever.

		Duration of Fever		
	Total	5–7 Days	8–14 Days	15–20 Days
Number of samples tested (1~3)	88	23	54	11
Detection method	Number of positive samples (%)			
1. IgM ELISA	61 (69.3%) *	14 (60%)	39 (72.2%) *	8 (72.2%) *
2. Nested PCR-serum samples	26 (29.5%)	10 (43.5%)	14 (25.9%)	2 (18.2%)
3. Nested PCR-urine samples	21 (23.9%)	7 (30.4%)	12 (22.2%)	2 (18.2%)
4. Bacterial culture <No. tested>	4 < 20 > (20%)	1 < 4 > (25%)	3 < 15 > (20%)	0 < 1 > (0%)

* *p* < 0.01, compared with nested PCR (serum sample)-positive rate.

**Table 2:** Sociodemographic characteristics of leptospirosis patients (*n* = 75) and non-patients (*n* = 42).

Variable	Category	Number of Patients (*n* = 75)	Number of Non-Patients (*n* = 42)	*p*-Value
Gender	Male	70 (93.3%)	31 (73.8%)	0.005
	Female	5 (6.7%)	11 (26.2%)	
Age	0–15 years	2 (2.7%)	1 (2.4%)	1.000
	>15–30 years	25 (33.3%)	12 (28.6%)	0.681
	>30–45 years	24 (32%)	20 (47.6%)	0.113
	>45–60 years	21 (28%)	3 (7.1%)	0.008
	>60	3 (4%)	6 (14.3%)	0.068
Residence area	Rural	69 (92%)	38 (90.5%)	0.745
	Urban	6 (8%)	4 (9.5%)	
Education level	No education	22 (29.3%)	8 (19.1%)	0.273
	Primary education	29 (38.7%)	20 (47.6%)	0.435
	Secondary education	17 (22.7%)	9 (21.4%)	1.000
	Higher education	7 (9.3%)	5 (11.9%)	0.754
Occupation	Farmer	39 (52%)	20 (47.6%)	0.702
	Housewife	7 (9.3%)	9 (21.4%)	0.092
	Day labourer	8 (10.7%)	1 (2.4%)	0.154
	Businessman	5 (6.7%)	3 (7.1%)	1.000
	Student	4 (5.3%)	3 (7.1%)	0.700
	Fisherman	4 (5.3%)	0 (0%)	0.295
	Retired	3 (4%)	2 (4.8%)	1.000
	Others	6 (8%)	3 (7.1%)	1.000
Socioeconomic condition	Poor	68 (90.7%)	34 (81%)	0.155
	Middle	7 (9.3%)	8 (19%)	0.155
	Higher	0 (0.0%)	0 (0%)	1.000
Onset (2024)	June	1 (1.3%)	0 (0%)	1.000
	July	8 (10.6%)	4 (9.5%)	1.000
	August	15 (20%)	8 (19%)	1.000
	September	29 (38.7%)	7 (16.7%)	0.021
	October	18 (24%)	18 (42.9%)	0.039
	November	4 (5.3%)	5 (11.9%)	0.279

**Table 3:** Clinical features and laboratory findings in leptospirosis patients.

Variable	Category	Frequency	%
Clinical findings (*n* = 75)	Fever	75	100
	Malaise	72	96
	Jaundice	63	84
	Nausea/Vomiting	63	84
	Headache	52	69.3
	Abdominal Pain	51	68
	Myalgia	50	66.7
	Cough	33	44
	Oliguria	32	42.7
	Calf Muscle Pain	27	36
	Back Pain	24	32
	Diarrhea	23	30.7
	Shortness of Breath	22	29.3
	Disorientation	17	22.7
	Joint Pain	16	21.3
	Conjunctival Suffusion	11	14.7
	Edema	11	14.7
	Rash	7	9.3
	Hemoptysis	3	4
	Neck Stiffness	3	4
Blood count findings (n = 57)			
Leukocytosis	>11,000/mm^3^	37	64.9
Neutrophilia	>75.0%	39	64.9
Lymphopenia	<20.0%	44	77.2
Thrombocytopenia	<150,000/mm^3^	44	77.2
Low Hemoglobin	<12 mg/dL	39	68.4
Raised ESR	Male ≤ 10 mm/h, Female ≤ 20 mm/h	57	100
Biochemical findings			
Raised serum bilirubin (*n* = 45)	>1.2 m/dL	42	93.3
Raised serum creatinine (*n* = 54)	>1.2 m/dL	43	79.6
Raised ALT (*n* = 47)	>45 U/L (male), >34 U/L (female)	32	68.1
Hyponatremia (*n* = 42)	<135 mmol/L	30	71.4
Hypokalemia (*n* = 42)	<3.5 mmol/L	12	28.6
Hypochloremia (*n* = 42)	<98 mmol/L	12	28.6
Prolonged Prothrombin Time (*n* = 16)	>13 s	14	87.5
Urine examination (*n* = 41)	Pus cell (+)	34	82.9
	Proteinuria	12	29.3
	RBC (+)	9	21.9
Ultrasound findings (*n* = 27)	Nephritis	17	63
	Hepatitis	11	40.7
	Ascites	7	25.9
	Hepatomegaly	6	22.2
	Splenomegaly	6	22.2

**Table 4:** Risk factors of leptospirosis patients living in rural and urban areas (*n* = 75).

Variable	Category	Total (*n* = 75)	Rural (*n* = 69)	Urban (*n* = 6)	*p*-Value
Exposure to water and soil	Sewage water	6 (8.0%)	3 (4.3%)	3 (50.0%)	0.005
	River	14 (18.7%)	14 (20.3%)	0 (0.0%)	0.586
	Standing water	54 (72.0%)	52 (75.4%)	2 (33.3%)	0.048
	Mud/wet soil	56 (74.7%)	54 (78.3%)	2 (33.3%)	0.033
Participation	Working in a paddy field	39 (52.0%)	39 (56.5%)	0 (0.0%)	0.010
	Fishing	28 (37.3%)	27 (39.1%)	1 (16.7%)	0.401
	Swimming	25 (33.3%)	23 (33.3%)	2 (33.3%)	1.000
	Recreational water activities	2 (2.7%)	0 (0.0%)	2 (33.3%)	0.005
Exposure to animals	Cattle	52 (69.3%)	52 (75.4%)	0 (0.0%)	0.001
	Dog	51 (68.0%)	51 (73.9%)	0 (0.0%)	0.001
	Cat	36 (48.0%)	35 (50.7%)	1 (16.7%)	0.202
	Rodents	47 (62.7%)	45 (65.2%)	2 (33.3%)	0.188
Heavy rain	Yes	65 (86.7%)	61 (88.4%)	4 (66.7%)	0.180
Wound history	Yes	6 (8.0%)	6 (8.7%)	0 (0.0%)	1.000
Travel history	Yes	13 (17.3%)	10 (14.5%)	3 (50.0%)	0.061

**Table 5:** Major species of *Leptospira* in humans in South and Southeast Asia reported in representative studies.

Country	Year	Dominant Species *^1^ (Serovar *^2^)	Reference
Philippines	2017–2018	*L. interrogans*	[42]
Thailand	2000–2005	*L. interrogans*, *L borgpetersenii, L. kirschneri*	[43]
	2001–2002, 2011–2012	*L. interrogans* (Autumnalis)	[44]
Lao PDR	2006–2007	*L. interrogans*, *L. weilii*, *L borgpetersenii, L. kirschneri*	[45]
Viet Nam	2018–2019	*L. interrogans* (Icterohaemorrhagiae, Wolffi, Hebdomadis)	[46]
Malaysia	2013	*L borgpetersenii* (Sejroe, Javanica), *L. interrogans* (Pyrogenes, Icterohaemorrhagiae), *L. weilii* (Celledoni)	[47]
	2016–2017	*L. interrogans*, *L. kirschneri*, *L. wolffii*	[30]
	2004–2020	*L. interrogans*, *L. kirschneri*, *L. wolffii*, *L. weilii*	[48]
India	2000–2010	*L. interrogans* (Tarassovi, Australis)	[49]
	1994–2009	*L. interrogans*, *L borgpetersenii, L. kirschneri, L. wolffii*	[29]
	2021	*L. interrogans* (Bataviae, Australis)	[50]
Sri Lanka	2016–2019	*L. interrogans*, *L borgpetersenii, L. weilii, L. kirschneri*	[51]

*^1^ Underline indicates a major species (serovar) described. *^2^ Serovars are shown in parentheses with presumptive species, only when a serological test (MAT) was performed.

## Data Availability

The data presented in this study are available from the corresponding author upon reasonable request, due to ethical restrictions on data sharing.

## Supplementary Materials

The following supporting information can be downloaded at: https://www.mdpi.com/article/10.3390/tropicalmed10100290/s1, Table S1: Selected *Leptospira* samples sequenced for 16S rRNA gene; Table S2: Nucleotide sequence identities (%) of partial 16S rRNA among *Leptospira* samples; Table S3: Nucleotide sequence identities (%) of partial 16S rRNA among *Leptospira* samples in the present study and previously identified strains in Bangladesh, the prototype strain (Khorat2), and worldwide circulating strains (Malaysia, Ecuador, and Iran).
