# Supplementary material for: Endemicity, Clinical Features, Risk Factors, and the Potential for Severe Infection in Leptospira wolffii-Associated Leptospirosis in North-Central Bangladesh
Source: Trop Med Infect Dis. 2025 Oct 13;10(10):290. doi: 10.3390/tropicalmed10100290 (PMC12568162; doi:10.3390/tropicalmed10100290)
Supplement: Supplementary file 1 [file tropicalmed-10-00290-s001.zip › Lepto2025-TableS1.pdf]

Table S1 Selected *Leptospira* samples sequenced for 16S rRNA gene

Serial no.	Sample ID	Specimen type	Date of Collection
1	L.wolffii.Anika.MMC.BD.2024.1	Blood	July. 2024
2	L.wolffii.Anika.MMC.BD.2024.2	Blood	July. 2024
3	L.wolffii.Anika.MMC.BD.2024.11	Blood	July. 2024
4	L.wolffii.Anika.MMC.BD.2024.17	Blood	August, 2024
5	L.wolffii.Anika.MMC.BD.2024.22	Blood	August, 2024
6	L.wolffii.Anika.MMC.BD.2024.33	Blood	August, 2024
7	L.wolffii.Anika.MMC.BD.2024.35	Blood	September,2024
8	L.wolffii.Anika.MMC.BD.2024.40	Blood	September,2024
9	L.wolffii.Anika.MMC.BD.2024.42	Blood	September,2024
10	L.wolffii.Anika.MMC.BD.2024.57	Blood	September,2024
11	L.wolffii.Anika.MMC.BD.2024.59	Blood	September,2024
12	L.wolffii.Anika.MMC.BD.2024.69	Blood	October, 2024
13	L.wolffii.Anika.MMC.BD.2024.71	Blood	October, 2024
14	L.wolffii.Anika.MMC.BD.2024.85	Urine culture	October, 2024
15	L.wolffii.Anika.MMC.BD.2024.88	Urine culture	October, 2024
16	L.wolffii.Anika.MMC.BD.2024.106	Urine culture	October, 2024
17	L.wolffii.Anika.MMC.BD.2024.107	Urine culture	October, 2024
